# Multiple Targets for Novel Therapy of FSGS Associated with Circulating Permeability Factor

**DOI:** 10.1155/2017/6232616

**Published:** 2017-08-10

**Authors:** Virginia J. Savin, Mukut Sharma, Jianping Zhou, David Genochi, Ram Sharma, Tarak Srivastava, Amna Ilahe, Pooja Budhiraja, Aditi Gupta, Ellen T. McCarthy

**Affiliations:** ^1^Renal Research Laboratory, Research and Development, MBRF and Kansas City VA Medical Center, Kansas City, MO, USA; ^2^Kidney Institute, University of Kansas Medical Center, Kansas City, KS, USA; ^3^Section of Nephrology, Children's Mercy Hospital and University of Missouri at Kansas City, Kansas City, MO, USA

## Abstract

A plasma component is responsible for altered glomerular permeability in patients with focal segmental glomerulosclerosis. Evidence includes recurrence after renal transplantation, remission after plasmapheresis, proteinuria in infants of affected mothers, transfer of proteinuria to experimental animals, and impaired glomerular permeability after exposure to patient plasma. Therapy may include decreasing synthesis of the injurious agent, removing or blocking its interaction with cells, or blocking signaling or enhancing cell defenses to restore the permeability barrier and prevent progression. Agents that may prevent the synthesis of the permeability factor include cytotoxic agents or aggressive chemotherapy. Extracorporeal therapies include plasmapheresis, immunoadsorption with protein A or anti-immunoglobulin, or lipopheresis. Oral or intravenous galactose also decreases *P*_alb_ activity. Studies of glomeruli have shown that several strategies prevent the action of FSGS sera. These include blocking receptor-ligand interactions, modulating cell reactions using indomethacin or eicosanoids 20-HETE or 8,9-EET, and enhancing cytoskeleton and protein interactions using calcineurin inhibitors, glucocorticoids, or rituximab. We have identified cardiotrophin-like cytokine factor 1 (CLCF-1) as a candidate for the permeability factor. Therapies specific to CLCF-1 include potential use of cytokine receptor-like factor (CRLF-1) and inhibition of Janus kinase 2. Combined therapy using multiple modalities offers therapy to reverse proteinuria and prevent scarring.

## 1. Introduction

Nephrotic syndrome is defined by loss of protein in the urine, decreased plasma albumin, fluid retention resulting in edema formation, and, in many cases, hyperlipidemia. The discovery of mutations in nephrin as the genetic cause of proteinuria that characterizes Finnish nephropathy [1] led to the understanding that nephrotic syndrome is the result of glomerular podocyte dysfunction. Persistent proteinuria is a precursor of renal scarring and decline in glomerular filtration and may, indeed, be a cause of progression of renal disease. Mutations affecting the structure or function of podocytes or their response to injury may lead to proteinuria and to glomerular scarring. However, currently identified monogenic disorders account for a minority of cases [2], and the cause of podocyte dysfunction or loss leading to nephrotic syndrome is not known in most patients. Glomerular scarring may affect only certain segments of a minority of glomeruli in a pattern termed focal and segmental glomerulosclerosis (FSGS). FSGS is an orphan disease representing a syndrome with many causes [3] and is the cause of about 3.3% of end-stage-renal disease in the United States [4]; it occurs worldwide and affects all races and ethnic groups. The frequency of FSGS appears to be increasing worldwide [5]. Certain patients with renal failure due to FSGS experience recurrence of proteinuria and of progressive renal dysfunction after renal transplantation [6]. This review will focus on the rationale for therapies and on trials that address mechanisms that may reverse proteinuria and prevent or delay renal failure in patients with nephrotic syndrome and features of FSGS. Such therapies may be useful in treatment of idiopathic nephrotic syndrome (INS) and in the treatment of recurrent FSGS in renal allografts.

## 2. Mutations That Point to Therapeutic Targets

Analysis of the mutated proteins in familial or monogenetic FSGS indicates several podocyte functions that render them vulnerable to injury or loss. Some are structural podocyte proteins including those of the slit-diaphragm (nephrin, Neph1), actin associated proteins (alpha-actinin 4) [11], CD2AP [12], and podocin [13]. Others are components of the glomerular basement membrane (GBM) [14, 15] and of the attachment complex including integrins [16] that provide physical adherence as well as “outside-in” signaling that maintain differentiated function. Still others are channels such as TRPC6, a gated calcium channel [17], mutations of which may predispose to injury mediated by increased magnitude and duration of signals by ANG II and other humoral responses. Finally, mitochondrial disorders lead to impaired energy availability and cellular damage [18]. APOL 1 risk mutations lead to increased incidence and rapid progression of FSGS in certain African Americans [19]. Each of these mutations leads to potential for therapies to protect or stabilize podocyte function and to minimize injury to the filtration barrier and consequent glomerulosclerosis. Current technology permits testing for these and other mutations [20]. The study of a cohort of patients with nephrotic syndrome is ongoing [21] and new point mutations associated with FSGS continue to be identified from whole genome sequencing [2].

## 3. Clinical and Experimental Evidence for a Circulating Factor

Evidence for the presence of a circulating factor that causes glomerular or podocyte injury and proteinuria includes early onset of proteinuria after renal transplantation in patients with FSGS [22], occurrence of proteinuria in infants of mothers with FSGS [23], transmission of proteinuria to rats or mice by FSGS plasma or fractions thereof [24, 25], remission after plasmapheresis [26, 27] or immunoadsorption with protein A [28] or anti-human IG [29] columns, and recovery of proteinuria in a transplanted kidney after retransplantation into a patient without FSGS as his underlying disease [30]. NS in each of these settings is initiated and perpetuated by factor or factors that are extrinsic to the kidney but intrinsic to blood that is perfusing the kidney. The identity of this substance has not been proven.

## 4. Role of Lymphocytes in NS

Lymphocytes have been implicated in the etiology of INS/FSGS on the basis of clinical observations and response to therapy and several experimental models in mice and rats. The initial postulate that T lymphocytes are the source of a substance causing proteinuria in INS was put forward by Shalhoub in 1974 [31]. This theory had no experimental basis but was derived from clinical observations including the lack of evidence of a humoral antibody response and remission induced by measles, steroids, or cyclophosphamide. The observations that lymphoma was sometimes accompanied by NS and that therapy resolved proteinuria reinforced the potential causal relationship. Studies of lymphocyte-derived products have failed to identify any unique etiological substance in INS. Glucocorticoids remain in use as first-line agents in INS and the severity of renal disease in INS is often defined by the responsiveness to corticosteroids. Steroid resistant or steroid dependent INS is more likely to progress to pathological FSGS and to renal failure than is steroid sensitive INS. ACTH, first introduced in the 1950s, has returned as ACTH gel (H.P. Acthar Gel, Mallinckrodt Pharmaceutical) [32, 33] It appears to have efficacy that may exceed that of glucocorticoids, perhaps because of interaction of the melanocortin receptor, MC1R [33–35]. B cells have also been implicated by the efficacy in some human trials of rituximab or similar monoclonal antibodies that target CD40 on B cells and podocytes

A model of FSGS in which glomerular damage appears to be mediated by abnormal T cells is that of the Buffalo/Mna rat. Rats develop proteinuria by 8 weeks of age. There is accompanying macrophage activation and expression of Th2 lymphocyte markers. Kidneys transplanted into affected rats develop glomerular injury. Investigators postulated that a circulating proteinuric substance might be present in both native and transplanted kidneys but have not identified this substance [36, 37]. An additional model of MCNS was derived by injecting CD34(+) stem cells or CD34(−) peripheral blood mononuclear cells from afflicted patients into immunocompromised mice. Both CD34(+) and CD34(−) cells lead to engraftment of human CD45(+) leukocytes but only CD34(+) cells induced albuminuria. Glomeruli from proteinuric mice showed podocyte foot process effacement. Authors concluded that cells responsible for INS are likely to be immature differentiating cells rather than mature peripheral T cells [38]. No follow-up studies using this model have been published.

Participation of B cells in FSGS is suggested by the success of anti-B cell antibodies such as Rituximab in FSGS therapy as well as by clinical responses to immunoadsorption using protein A [39] or anti-human IgG [40]. Although there is no antibody staining in FSGS or its recurrence, a survey of sera from patients with recurrent FSGS contained a large number of autoantibodies compared to non-FSGS patients (7800 antibodies versus 780 in patients with other renal diseases). Ten proteins were selected from those represented in the kidney to make up a panel for testing as a marker for recurrence of FSGS after transplantation [41]. Antibodies included anti-CD40, PTPRO, CGB5, FAS, P2RY11, SNRPB2, and APOL2. Positive results using this panel had a high predictive value of posttransplant recurrence in a retrospective cohort of 64 transplanted FSGS patients. Anti-CD40 was the most highly related to recurrent FSGS. Authors presented evidence that suPAR and anti-CD40, given together in mice, produced significant proteinuria and that blocking activation of *α*v*β*3 integrin interfered with this proteinuria. No further studies regarding this interaction have been published but its relevance and the potential for future clinical trials has been proposed in a recent review [42]. We have observed that a monoclonal antibody to PTPRO increases *P*_alb_ during in vitro testing [43].

Bone marrow transplantation has been used to decrease the incidence of recurrence after renal transplantation; the optimal use of this strategy has not been established [44]. We have reported that *P*_alb_ activity and proteinuria were abolished for more than 10 years in a patient who was treated with standard therapy for Hodgkin's lymphoma [45]. This case report illustrates the potential role of aggressive cytotoxic therapy in eliminating circulating permeability factor or factors.

## 5. Lessons from In Vitro Studies of Isolated Glomeruli and Cultured Podocytes

**Figure 1:**
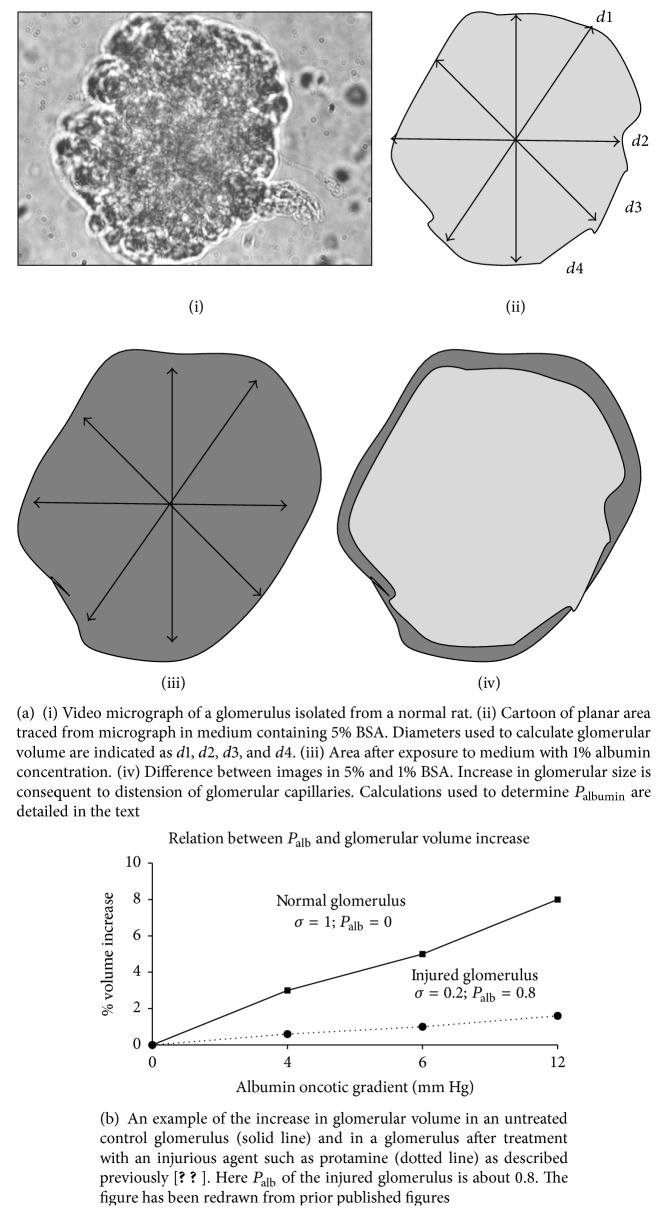


Additional information about renal responses in FSGS has been derived from studies of isolated glomeruli or of cultured podocytes. We and others have incubated glomeruli from experimental animals with plasma or sera from individuals with INS or FSGS or with other noninflammatory renal diseases [8, 46, 47]. The original assay is based on measurement of capillary expansion after filtration across the capillary wall in response to an albumin oncotic gradient. Convectional albumin permeability of the glomerular capillary barrier is calculated from the increase in glomerular volume as shown in Figure 1(a). Calculation is as follows [7]. Glomerular volume in each condition is calculated from the geometric mean of 4 diameters at 45° angles as indicated using the formula: *V* = (4/3)*π*(*d*/2)^3^. Volume increase is calculated as follows: Δ*V* = *V*_1%_ − *V*_5%_. Since Δ*V* ∝ Δoncotic  pressure × *σ*_albumin_, *σ*_albumin control_ = 1, *σ*_albumin experimental_ can be calculated as follows: Δ*V*_experimental_/Δ*V*_control_. We have defined albumin permeability after experimental treatment from the formula: *P*_albumin experimental_ = (1 − *σ*_albumin experimental_) or (1 − Δ*V*_experimental_/Δ*V*_control_). *P*_albumin_ or *P*_alb_ is a dimensionless parameter which ranges from zero in the normal glomerular capillary to a maximum of 1.0 after injury.

We have used the assay to define an activity level, termed “*P*_alb_ activity” or simply “*P*_alb_” [48]. We and our colleagues have recently reported the use of a comparable method in which fluorescent albumin is used as a marker for filtration and for measuring *P*_alb_ [49]. *P*_alb_ testing has been standardized using normal or patient plasma in a 1 : 50 dilution and an oncotic gradient of 16–20 mm Hg [8]. Sera of children with INS cause moderate damage to the glomerular protein filtration barrier [47] while sera of individuals with collapsing FSGS [46] or from patients with posttransplant recurrence of FSGS caused a profound loss of barrier function [8]. The assay is reliable across a range of observers. The correlation coefficient between values of replicate samples from 35 patients was 0.72 (*p* < 0.001) [8] and have been stable and reproducible in studies of human samples and the original assay and its modifications have been employed in a wide range of experimental protocols for more than 30 years in several centers [49–51].

Studies of the effects of plasma or serum from patients with FSGS on isolated glomeruli have documented a profound and nearly immediate increase in *P*_alb_. The increase is accompanied and is dependent on phosphorylation of JAK2 and STAT3 [9]. Comparable activation of the JAK/STAT pathway occurs in cultured immortalized podocytes incubated with FSGS serum [9]. The effect of patient serum is greatest in samples from patients with the most aggressive renal disease as indicated both by a more rapid course to ESRD [52] and by comparison of the very high *P*_alb_ activity of sera from patients with collapsing FSGS [46] or recurrence after transplantation versus those with MCNS or transplantation without recurrence [8]. *P*_alb_ activity predicts posttransplant recurrence in patients with podocin mutations [53]. *P*_alb_ activity is decreased or abolished by PP [8, 54] or IA with protein A [40]. Activity is blocked by JAK or STAT inhibitors [9], by the addition of galactose or adsorption of sera on a galactose column [55], or by a variety of potential therapeutic agents including indomethacin [56], cyclosporine A [57], NO, acting as a superoxide scavenger [58], antibody to CLCF-1 or by CRLF-1 [9], extract of Tripterygium wilfordii [59], and eicosanoids 20-HETE [60] and 8,9 EET [61]. One or more components from plasma of humans and other mammals [62, 63] and urine from patients with recurrent FSGS also block *P*_alb_ activity [64].

## 6. Proposed Candidates for the FSGS Permeability Factor

We and others have attempted to identify substance or substances in the plasma of patients with FSGS, especially those who are resistant to therapy or who progress to ESRD and experience recurrent nephrotic syndrome and FSGS after transplantation [25]. Historical candidates as proteinuric factors include vascular permeability factor (VPF) that is synthesized by T cells [65] and hemopexin [66]. More recently, elevated concentrations of soluble urokinase-type plasminogen activator receptor (suPAR) have been reported in patients with FSGS. This finding and results of studies of cultured podocytes have led to the proposal of suPAR as a lead candidate as an etiological factor in FSGS. Studies of cultured podocytes have been interpreted as supportive [67]. SuPAR effects include altered cytoskeleton in immortalized podocytes, altered podocyte attachment with activation of *β*3 integrin [68], and activation of STAT1 in vascular smooth muscle cells via a PDGFR receptor [69]. SuPAR has numerous other effects on several cell types including alteration of mobilization and migration and of mesenchymal stem cells [70]. Other investigators have disputed the interpretation of the experimental and clinical findings regarding suPAR [71, 72]. Active investigations are ongoing, but suPAR is elevated in many inflammatory states and in diabetes mellitus. SuPAR appears to be associated with progression of renal disease regardless of its etiology [73, 74]. Circulating suPAR is increased in renal failure in part because of diminished loss in urine [75]. Understanding of the role that suPAR plays in the initiation and progression of renal disease, including INS and FSGS, is incomplete.

A role for lipoproteins in FSGS is suggested by reports of successful therapy using lipopheresis and by the presence of abnormal lipids in proteomic analyses. In addition, lipoproteins have been proposed both as inhibitors of FSGS permeability activity and as candidates for the active factor. Specific apolipoproteins that act as inhibitors of *P*_alb_ activity of FSGS sera include APO-E2, APO-E4, and fragments of APO-A iv [76]. Genotyping of apolipoproteins did not reveal differences between FSGS patients who were sensitive to steroids and those who were resistant to therapy [77]. In contrast, a form of APO-A1 was identified in plasma only in recurring FSGS patients and was absent from those without recurrence [78]. Additional observations that implicate lipids in proteinuria are derived from work indicating the role of rituximab as an inhibitor of downregulation of podocyte sphingomyelinase-like phosphodiesterase 3b (SMPDL-3b) [79].

We have recently performed studies to enrich *P*_alb_ activity of recurrent FSGS plasma and have identified cardiotrophin-like cytokine-1 (CLCF-1) in the active fraction of FSGS plasma using tandem LC-MS/MS [10, 80]. CLCF-1 is best known for its action through the JAK/STAT pathway. CLCF-1 is a member of the IL-6 family of cytokines. CLCF-1 was initially cloned from T cells [81, 82] and is a neurotrophic and B cell stimulating factor that increases expression of immunoglobulins. CLCF-1 acts through a complex receptor composed of CNTFR, LIFR, and gp130 [83]. Gp130 defines the IL-6 family of cytokines while, in the case of CLCF-1, CNTFR and LIFR confer specificity [84]. The IL-6 family includes a number of pleiotropic cytokines which are expressed by both bone marrow derived and somatic cells. The IL-6 family includes erythropoietin, Leukemia Inhibitory Factor (LIF), and Ciliary Neurotrophic Factor (CNTF), as well as IL-6 itself. CLCF-1 has 3 potential binding sites and we have shown that activation of podocytes is prevented interaction with CRLF-1 [9], a related and cosecreted cytokine [85]. Receptor-ligand binding initiates signaling by the JAK/STAT pathway. We have reported that podocytes express primarily JAK2 and STAT3 [10] and each is activated by tyrosine phosphorylation by CLCF-1 at specific sites. STAT3 activation of peripheral blood cells is immediate and returns to normal within 24 hours [10]. In contrast, STAT3 phosphorylation in the renal cortex persists for at least 72 hours [10]. pSTAT3 is present in glomeruli at a location consistent with podocytes as well as in vascular smooth muscle within the kidney and proximal tubule cells [10]. CLCF-1 also activates STAT3 in cultured murine or human podocytes and simultaneously alters the actin cytoskeleton [10]. These changes may be analogous to those required for foot process retraction in vivo during proteinuric states.

**Figure 2:**
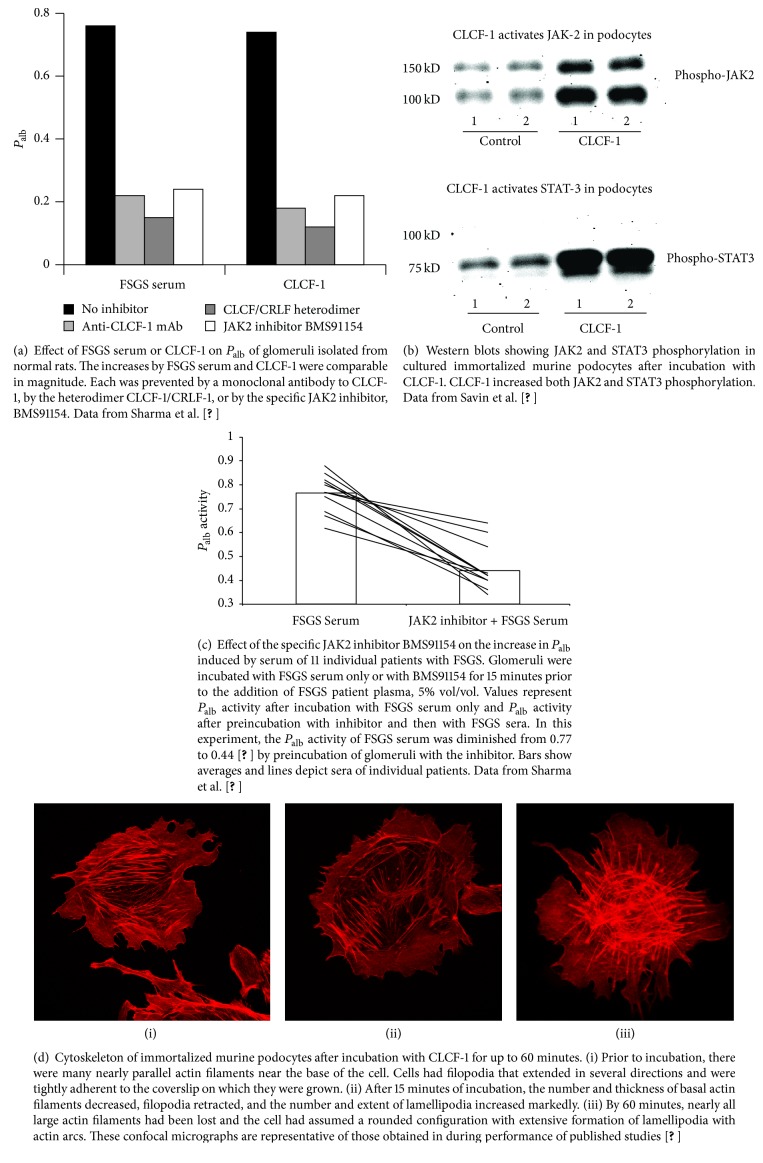


We have shown that *P*_alb_ activity is removed by plasmapheresis (PP) with a kinetic pattern comparable to that of immunoglobulins and that activity can be recovered from discarded plasmapheresis fluid [8]. We have followed *P*_alb_ activity during sequential fractionation of FSGS plasma obtained by plasmapheresis [25, 86]. The active component of the plasma fraction is soluble in ammonium sulfate at a concentration of 70% but precipitates at 80%. Injection of this fraction into rats results in proteinuria [25]. In additional studies, we found that *P*_alb_ activity is retained on a column of galactose-coated agarose beads and can be recovered by elution using a galactose solution [55]. Activity is retained by a filtration membrane with molecular weight cut-off of 30 kDa. We have performed liquid chromatography/tandem mass spectrometry (LC/MS/MS) on the plasma fraction obtained by galactose affinity chromatography of FSGS plasma and have identified a candidate cytokine, cardiotrophin-like cytokine factor-1 (CLCF-1) [80]. This cytokine mimics the *P*_alb_ activity of FSGS plasma [9, 10] and a monoclonal antibody to CLCF-1 markedly decreases the activity of FSGS sera [9]. In addition, both FSGS serum and CLCF-1 activate the JAK2/STAT3 pathway [9]. *P*_alb_ activity of FSGS serum as well as that of CLCF-1 is inhibited by specific inhibitors of JAK2 and STAT3 [9] and by FDA approved JAK inhibitors ruxolitinib (Incyte), by tofacitinib (Pfizer), and by baricitinib (E. Lilly), which is in review by the FDA. *P*_alb_ activity and podocyte STAT phosphorylation are also inhibited by cytokine receptor-like factor-1 (CRLF-1), a cytokine which dimerizes with CLCF-1 [9]. The effects of CLCF-1 on *P*_alb_ and on JAK2 and STAT3 activation are shown in Figures 2(a) and 2(b).  Figure 2(c) illustrates that JAK2 inhibition blocks the effect of FSGS sera on *P*_alb_ activity of sera from FSGS patients (Figure 2 depicts data previously published [9, 10]). We are continuing to investigate the role of CLCF-1, CRLF-1, and related molecules in the genesis of proteinuria in FSGS in native kidneys and in posttransplant recurrence of FSGS using isolated glomeruli, cultured podocytes, and responses in intact rodents.

The question of design and interpretation of animal models for recurrent FSGS mediated by a circulating molecule or molecules has not been resolved. Proteinuria is a multistep process. The initial change in permeability is the result of podocyte signaling and a functional increase in permeability. Filtered albumin is reabsorbed by the proximal tubule and reabsorption may delay recognition of the altered filtration barrier. Visual confirmation of proximal tubule reabsorption is provided by 2-photon imaging of rat kidneys in several models of proteinuria [87]. Anatomical changes follow and include impaired podocyte architecture and attachment and to podocyte loss. Glomerular segmental scarring is evident only in later phases and leads to altered hemodynamics and rheology. It is proposed that loss of podocytes exposes glomerular basement membrane and permits movement of parietal epithelial cells to vacant basement membrane where they form an attachment [88] that alters the geometry of both the capillary and Bowman's space. As the disease advances, there is scarring in the tubular interstitial compartment with capillary loss, tubular atrophy, and collagen accumulation. Late in the process, local or circulating cells and soluble substances alter the interstitium leading to accumulation of extracellular material and interstitial fibrosis. The role of the circulating permeability factor in each of these processes is not known. In our opinion, many of the later events that lead to progressive renal failure are shared with other renal diseases.

We propose that models that are based on effects of toxins such as puromycin, adriamycin, bisphosphonates, or reduced nephron number [89] or on podocyte death induced by diphtheria toxin in genetically modified mice [90] or by antibodies to podocytes [91] are not suitable models for the early and reversible proteinuria of recurrence FSGS after transplantation. We have focused on the initiating steps of podocyte signaling and altered attachment rather than on processes that determine subsequent glomerular scarring and interstitial fibrosis. We believe that this focus may permit the design of preemptive treatment. To date, no animal model of glomerular dysfunction that arises directly from responses to a plasma substance and leads to renal failure has been defined. Targeted therapy may require knowledge of cell signaling during exposure to plasma of patients with recurrent FSGS or to the components of such plasma. Human trials will be required to prove the relevance and efficacy of proposed therapies.

Cultured immortalized murine and human podocytes have also been used in functional assays to define plasma activity. Assay parameters include alterations in the pattern of actin cytoskeleton [68]. Activation of *β*3 integrin, as evidenced by its phosphorylation, has recently been proposed as an indicator of podocyte injury and a high throughput assay presented as a model for discovery of novel therapeutics [92]. Increased podocyte motility manifest as migration into a “scratch” defect in a nearly confluent cell layer has also been used to measure activity [68, 93]. We and others have used alterations in cytoskeleton configuration as a measure of the effects of FSGS samples and confirmed that cytoskeleton is reorganized after incubation in FSGS serum or a candidate cytokine [10].  Figure 2(d) illustrates cytoskeleton responses of podocytes during incubation with CLCF-1. During incubation with CLCF-1, filopodia retract and basal parallel actin filaments are attenuated and decrease in number. Simultaneously, lamellipodia become more prominent, the cells assume a more rounded state, and short nonparallel actin filaments and actin arcs predominate [10].

## 7. Targets for Therapy in FSGS and Its Recurrence

Trials in FSGS and its posttransplant recurrence have focused on agents that may remove or neutralize the injurious factors themselves (PP, IA [28, 94–96], and galactose [55, 97–99]), modify the immune response by interacting with glucocorticoid and/or melanocortin receptors (prednisone, dexamethasone, and ACTH as Acthar Gel), alter cellular immunity (calcineurin inhibitors including tacrolimus, cyclosporine A, humanized antibodies to components of B cells (rituximab), and T cells (abatacept)), and attempt to limit fibrosis by antibodies to the cytokines TGF*β* and TNF*α*. A number of agents, including glucocorticoids and calcineurin inhibitors, exert protective effects on podocytes by changing protein expression profiles [100–103] and stabilizing actin cytoskeleton [104]. The antiproteinuric effects of angiotensin converting inhibitors and angiotensin receptor blockers (ARBs) are well known and appear to occur regardless of the etiology of proteinuria. Results of a randomized controlled trial of a blocker both of angiotensin and of endothelin receptor have been reported in abstract form H. Trachtman. Am Soc Nephrol, Annual Meeting, November, 2016, HI-OR06, and showed marked reduction of proteinuria in about 28% of FSGS on sparsentan versus 9% in those treated with irbesartan. Clinical trials related to FSGS are listed in https://ClinicalTrials.gov and include therapies related to corticosteroids and ACTH gel, inhibitors of angiotensin and endothelin receptors, immunosuppressive agents including calcineurin inhibitors and monoclonal antibodies, extracorporeal interventions using PP, IA, and lipopheresis, an inhibitor of sodium/glucose transporter 2 (SGLT2), and treatment with vitamin D and retinoids. Results of most of these trials have not yet been published. Others have shown a positive effect on proteinuria or disease progression in only a minority of subjects [105].

## 8. Completed Trials in Patients with FSGS in Native Kidneys

Large-scale consortia are necessary to the study of orphan diseases such as FSGS.* The Toronto Registry* has followed patients with common glomerular diseases, FSGS, membranous nephropathy, and IgA nephropathy and has generated a rich experience in regard to natural history and therapy. Studies established that partial as well as complete remissions lead to more stable renal function and prolonged renal survival. Cyclosporine decreases proteinuria in glomerular disease of several etiologies; CsA-induced nephrotoxicity can be minimized by use of lower doses. Mycophenolate (MMF) also induces remission in many patients with FSGS. In a trial comparing CsA to no CsA in FSGS in native kidneys, CsA was better in reducing remissions [106]. Serum *P*_alb_ activity measured in our lab was not reduced even during clinical remission of proteinuria [107]. This suggests that CsA directly protects glomeruli from the effects of continued presence of the FSGS permeability factor. Additional studies also showed clinical benefit from CsA treatment [108, 109]. Results of studies of isolated glomeruli and podocytes confirm that CsA protects the permeability barrier from the effects of FSGS serum by nonimmune mechanisms [57]. Protection of synaptopodin and of the actin cytoskeleton [104] may be consequent to inhibition of calcineurin activity.

The NIH-funded* FSGS Clinical Trial* examined the relative efficacy of 2 accepted therapies in 138 subjects randomly assigned to CsA or MMF plus dexamethasone. The relevant outcome was complete or partial remission of proteinuria within 1 year. The patients were selected from those with steroid resistant FSGS with relatively well preserved renal function at the time of recruitment. Each treatment regimen induced remission in about 30% of patients. About 10% patients in each treatment group developed renal failure or died during the 78-week study. MMF plus dexamethasone had more side effects while CsA transiently decreased GFR. GFR did not differ between the 2 groups at the end of the study [110]. The trial was limited by relatively advanced disease and small number of subjects and the short duration of therapy.

The Phase 2* FONT (Novel Therapies for Resistant FSGS)* trial compared usual care to oral administration of galactose or injections of anti-TNF alpha (adalimumab, “Humira®”). As in the prior FSGS trial, patients had steroid resistant FSGS with GFR ≥ 40 ml/min/1.75 m^2^. The study was limited by the small number of enrolled subjects (21 subjects). No subject in the usual care arm had remission of proteinuria; 2 patients in each experimental treatment arm had remission. The effect of galactose was more prolonged and had higher patient acceptance than was seen with adalimumab treatment [99, 110]. An additional trial of galactose in glomerular disease [105] has been published as have several case reports [97, 98]. The trial was marred by heterogeneity of diagnosis and small number of subjects [105]. Case reports may have been subject to positive publication bias.

## 9. Trials to Prevent or Treat Transplant Recurrence of FSGS

A single center study of the use of plasmapheresis to prevent of delay FSGS recurrence after transplant was carried out in patients judged to be at high risk because of prior recurrence or rapid course to renal failure. There was an apparent benefit in preventing recurrence and early transplant loss [111]. The small size of the study and the lack of a control group limit the strength of the findings. Therapies used in other small series have failed to prevent recurrence.

## 10. Rituximab to Prevent Recurrence of Proteinuria in Patients Receiving Kidney Transplant for FSGS

An NIH sponsored single center clinical trial in preventing recurrence by the use of rituximab is being conducted. The underlying hypothesis is that rituximab will be protective because of its role in control of activity of podocyte sphingomyelinase-like phosphodiesterase 3b [79]. Rituximab partially prevented SMPDL-3b and ASMase downregulation that was observed in podocytes treated with the sera of patients with recurrent FSGS. Overexpression of SMPDL-3b or treatment with rituximab was able to prevent disruption of the actin cytoskeleton and podocyte apoptosis induced by patient sera. Subjects have been recruited and randomized but study results are not available at this time.

## 11. Individualized Immunoadsorption (IA) for Posttransplant Therapy

Reversal of recurrence and long-term graft survival has been reported in 66% of 18 renal transplant recipients with ESRD due to idiopathic FSGS. This report includes noteworthy details regarding the time course of recurrence. 66.7% of patients experienced disease recurrence in a mean time of 0.75 months after transplantation (KTx), with a mean proteinuria of 8.9 g/day at the time of recurrence. The majority of patients were adults (mean age, 30.8 years). Both cadaveric and living related donors were included. Four of the patients received therapy with rituximab in addition to IA. During a mean time of follow-up of 48.3 months, about 60% of patients achieved complete remission, and about 40% achieved partial remission during average follow-up of over 4 years. At the end of follow-up, 67% had functioning grafts and were in sustained remission, while 33% progressed to ESRD because of FSGS recurrence [95]. These results reinforce the notion that use of current therapies in an individualized and persistent manner can result in marked improvement in allograft survival.

## 12. Glomerular Disease after Kidney Transplant and Current and Future Clinical Trials

Although several agents have been proposed as mediators of FSGS recurrence after transplantation, none have been definitively proven as etiologic agents. Most studies regarding recurrence in this orphan disease have been descriptive. A study of 1435 adult kidney transplants at the Mayo Clinic showed that as many as 26% of recipients developed glomerular disease by 10 years. FSGS was the diagnosis in 38% of these while other diagnoses included IgA nephropathy, membranous nephropathy, and mesangial proliferative glomerulonephritis. The risk of glomerular disease was increased with younger age, females, and steroid free induction and lower pretransplant serum albumin. Recurrent FSGS increased the risk of graft failure (*R* = 2.82  *p* < 0.0001). Overall glomerular disease caused 22% of allograft losses. These findings emphasize the important impact of glomerular disease in the overall success and benefit of renal transplantation [112].

An antibody to CD80, abatacept, has been used to treat FSGS recurrence and some success was initially reported [113] but others have failed to confirm this effect [114]. Thus, the utility of this agent is not clear.

We have recently proposed a trial of the JAK inhibitor ruxolitinib (Jakifi, Incyte, Corp) (REFOCUS, Rescue FSGS). The decision to emphasize study patients with recurrent proteinuria and FSGS in renal allografts is based on the potential to identify the initial stages of injury at a time when there is little anatomic distortion and to provide intervention when the injury is most likely to be reversible. The strategy of enrolling only patients with recurrence will also minimize the likelihood of confusion arising from the presence of mutations of podocyte proteins and of inclusion of renal disease secondary to hypertension, obesity, or environmental toxins. The choice of ruxolitinib is based on its relative specificity for JAK1 and JAK2 [115]. As an FDA approved drug, its pharmacokinetics and toxicity have been well defined in both preclinical and clinical studies [116, 117]. In addition, it has been used in thousands of patients with hematological disorders including myelofibrosis [118] and polycythemia vera and it is under investigation in other hematological malignancies and solid tumors. Ruxolitinib and several other JAK inhibitors block the effects of FSGS sera on pSTAT in cultured podocytes.

We also plan to propose a formal trial of oral or intravenous galactose in a sample of FSGS patients that is sufficiently large to confirm or deny general efficacy in FSGS after transplantation and/or idiopathic FSGS in native kidneys. The selection of galactose is based on in vitro studies, on pharmacological effects of galactose on plasma activity, and on several case reports in pre- and posttransplant patients [55, 97–99, 105].

**Figure 3:**
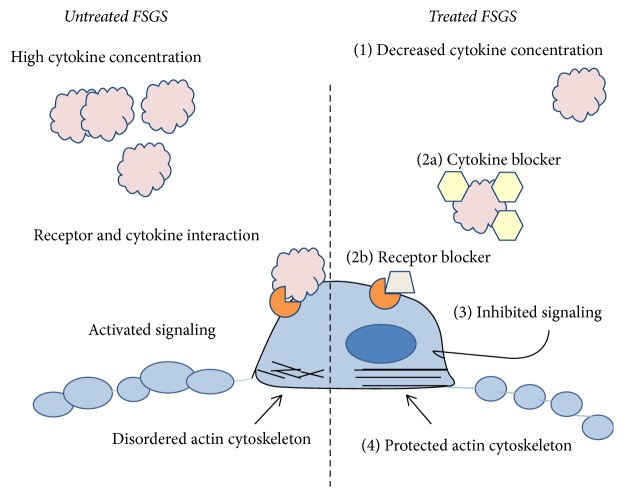
*Scheme showing potential targets for therapy in glomerular injury caused by circulating factor or cytokine*. The left panel shows the milieu that favors podocyte dysfunction and proteinuria. Excess activity of an injurious factor or cytokine permits its interaction with receptors on podocytes which, in turn, activates signaling via JAK2/STAT3 and other pathways. Actin cytoskeleton becomes disordered and podocyte architecture and function is altered. The right panel shows some potential treatment goals including the following: (1) decrease factor synthesis or remove it by plasmapheresis, immunoadsorption or other extracorporeal methods; (2a) administer blocker such as galactose or (2b) receptor blocker such as antibody to specific component or receptor; (3) inhibit intracellular signaling by JAK or STAT inhibitor or inhibitor of other essential cell pathways; (4) protect actin cytoskeleton by calcineurin inhibitors such as CsA or by a sphingomyelinase inhibitor such as rituximab. Identification of multiple targets will permit concurrent use of several modalities that may increase effectiveness while limiting side effects.

The available data regarding idiopathic FSGS is consistent with the concepts shown in Figure 3. As illustrated, a circulating substance, which has not been definitively defined and is labelled here as “cytokine,” is present in the circulation. This substance appears to interact with specific receptors on the podocytes, activating intracellular signaling pathways and leading to alterations in cytoskeleton, adhesion, and motility. Decreasing its total concentration by inhibiting its synthesis or increasing catabolism, blocking its activity by antibodies or inhibitors of receptor-ligand interaction, interrupting intracellular signaling, or enhancing cell mechanisms that protect cytoskeleton and adhesion may be developed as therapeutic interventions. Multicenter collaborative studies are essential since no center will have enough patients to permit meaningful analysis of treatment effect. Early intervention will provide an opportunity to arrest the disease in its initial stages. New therapies offer to dramatically improve the lives of patients with FSGS in native kidneys and permit successful renal transplantation in those who have progressed to end-stage-renal disease.

## 13. Summary

Studies of genes expressed in podocytes and the glomerular capillary wall point to cytoskeleton, cell junctions, cell attachment, and metabolism as potential targets for intervention. We and others have demonstrated that FSGS serum or plasma induces increased glomerular capillary permeability in vitro and alters cytoskeleton and signaling responses of cultured podocytes. These responses may be used to screen potential therapeutic agents prior to or simultaneously with conduct of clinical trials. A number of trials are ongoing in patients with FSGS in native kidneys or in recurrence after transplantation while others have been proposed and await implementation. Problems that must be overcome include heterogeneity of patients, short duration of studies, small sample size, and late initiation of therapy. Careful design and participation of consortia will be required to attain clinical and statistical significance.

## Abbreviations

8,9 EET: 8,9-Epoxyeicosatrienoic acid
20 HETE: 20-Hydroxyeicosatetraeonic acid
ACTH: Adrenocorticotrophic hormone
CLCF-1: Cardiotrophin-like cytokine factor-1
CNTF: Ciliary Neurotrophic Factor
CNTFR*α*: Ciliary Neurotrophic Factor Receptor *α*
sCNTFR*α*: Soluble Ciliary Neurotrophic Factor Receptor *α*
CRLF-1: Cytokine receptor-like factor-1
CsA: Cyclosporine A
FSGS: Focal segmental glomerulosclerosis
gp130: Glycoprotein 130
IA: Immunoadsorption
IL-6: Interleukin-6
INS: Idiopathic nephrotic syndrome
JAK: Janus kinase
LIFR*α*: Leukemia inhibitory factor receptor *α*
NS: Nephrotic syndrome
*P*_alb_: Glomerular albumin permeability, measured during in vitro studies
PP: Plasmapheresis
STAT: Signal transducer and activator of transcription
suPAR: Soluble urokinase-type plasminogen activator receptor
TGF*β*: Transforming growth factor-*β*
TNF*α*: Tumor necrosis factor-*α*
Tyk: Tyrosine kinase
VPF: Vascular permeability factor.
